# Excessive daytime sleepiness and migraine: a population-based study

**DOI:** 10.1186/1129-2377-14-S1-P7

**Published:** 2013-02-21

**Authors:** BK Kim, JM Kim, KS Lee, MK Chu

**Affiliations:** 1 Department of Neurology, Eulji University School of Medicine, Korea, Republic of; 2 Department of Neurology, Chungnam National University, College of Medicine, Korea, Republic of; 3 Department of Neurology, Seoul St.Mary's Hospital, The Catholic University of Korea, Korea, Republic of; 4 Department of Neurology, Hallym University College of Medicine, Korea, Republic of

## Background

Excessive daytime sleepiness is a common symptom, with a prevalence of 10-20% in general population and is reported to be with associated with migraine. However, the prevalence, clinical features, and impact of excessive daytime sleepiness among migraneurs in population-based setting have only rarely been reported.

## Objective

To assess the influence of excessive daytime sleepiness on clinical features and impact of migraine.

## Methods

We selected a stratified random population sample of Koreans over age 19 and evaluated them with a 60-item semi-structured interview designed to identify headache type using ICHD-2 criteria. We assessed the Epworth sleepiness scale (ESS) for assessing sleepiness and excessive daytime sleepiness (EDS) was defined as ESS ≥10. We also included items of HIT-6 to assess impact of headache.

## Results

Of 2,836 all participants, 152 (5.1%) were diagnosed as having migraine. EDS was more prevalent among migraineurs comparing to non-migraine controls (25.7% for migraineurs vs. 16.3% for non-migraine controls, p=0.003). Migraineurs with EDS reported higher attack frequency per month (7.0±9.7 attacks for migraineurs vs. 3.5±5.8 for non-migraine controls, p<0.000), higher VAS score for pain intensity (7.1±1.8 for migraineurs vs. 6.0±1.9 for non-migraine controls, p=0.006), and higher HIT-6 score (60.6±10.3 for migraineurs vs. 52.8±8.3 for non-migraine controls, p<0.000) comparing to migraineurs without EDS. Migraineurs with EDS showed more of depression (OR=5.67, 95% CI 2.5-12.7), insomnia (OR=2.98, 95% CI 1.1-8.4) and sleep disordered breathing (OR=2.78, 95% CI 1.1-7.3) than migraineurs without EDS. Unilateral pain, pulsating quality, aggravation by routine physical activity, nausea, vomiting, photophobia and phonophopbia were not significant according to EDS.

## Conclusions

EDS is prevalent among migraineurs in general population. Attack frequency, severity and impact by headache increase with EDS.
